# Acoustic structure and information content of trumpets in female Asian elephants (*Elephas maximus*)

**DOI:** 10.1371/journal.pone.0260284

**Published:** 2021-11-23

**Authors:** Evelyn Fuchs, Veronika C. Beeck, Anton Baotic, Angela S. Stoeger

**Affiliations:** Mammal Communication Lab, Department of Behavioral and Cognitive Biology, University of Vienna, Vienna, Austria; University Hospital Eriangen at Friedrich-Alexander-University Erlangen-Numberg, GERMANY

## Abstract

Most studies on elephant vocal communication have focused on the low-frequency rumble, with less effort on other vocalization types such as *the* most characteristic elephant call, the trumpet. Yet, a better and more complete understanding of the elephant vocal system requires investigating other vocalization types and their functioning in more detail as well. We recorded adult female Asian elephants (*Elephas maximus*) at a private facility in Nepal and analyzed 206 trumpets from six individuals regarding their frequency, temporal and contour shape, and related acoustic parameters of the fundamental frequency. We also tested for information content regarding individuality and context. Finally, we recorded the occurrence of non-linear phenomena such as bifurcation, biphonation, subharmonics and deterministic chaos. We documented a mean fundamental frequency ± SD of 474 ± 70 Hz and a mean duration ± SD of 1.38 ± 1.46 s (N_indiv._ = 6, N_calls_ = 206). Our study reveals that the contour of the fundamental frequency of trumpets encodes information about individuality, but we found no evidence for trumpet subtypes in greeting versus disturbance contexts. Non-linear phenomena prevailed and varied in abundance among individuals, suggesting that irregularities in trumpets might enhance the potential for individual recognition. We propose that trumpets in adult female Asian elephants serve to convey an individual’s identity as well as to signal arousal and excitement to conspecifics.

## Introduction

Elephants are highly social mammals that live in fission-fusion societies based on matriarchal female-bonded kin groups, from which males disperse when adolescent [1–8]. The ‘social complexity hypothesis’ for communication proposes that animals living in complex social systems do also require a more complex vocal communication system, consisting of structurally and functionally distinct elements [9]. Accordingly, elephants use a diverse set of vocalizations as part of their intra-specific communication. While the vocal repertoire has been well described for the African savanna elephant *Loxodonta africana* [10–14], fewer studies have been done on Asian elephants *Elephas maximus* [but see 15–17]. In-depth investigations on elephant vocalizations have mainly focused on low-frequency calls (rumbles) in African elephants (*Loxodonta* spp.) [18–26]. To gain a broader and better understanding of the elephants’ vocal communication system, further research on vocalizations other than the rumble, such as the characteristic trumpet, are much-needed, as all call types of the repertoire might convey important information and be crucial in specific behavioral contexts.

The trumpet in Asian and African elephants is generally produced in situations of high arousal [27]. African savanna elephants tend to trumpet when highly stimulated, in fearful, aggressive, playful or socially excited situations [14]. In African savanna elephants, Poole & Granli [28] and Poole [14] propose several trumpet types. This includes noisy/chaotic ‘nasal trumpets’ with underlying tonal structure, which are much noisier than ‘trumpets’ [14]. They further refer to different types of play trumpets: ‘harmonic-play-trumpets’, ‘noisy-play-trumpets’ and ‘pulsated-play-trumpets’, the latter being mainly associated with exuberant play (for example when running, which causes the pulsated structure). The trumpet of adult African savanna elephants has been described to have a mean fundamental frequency (F0) of 300–390 Hz with a duration of 0.7 up to 5 s [10, 11, 14]. In contrast, Sharma et al. [29] reported a mean F0 of about 600 Hz for trumpets of adult female Asian elephants and a difference in duration ± SEM depending on context; 0.43 ± 0.02 s in a disturbed context (induced by humans or other species) and 0.69 ± 0.06 s in an undisturbed state (contact calls or during social interaction). The acoustic structure of Asian elephant trumpets has also been described by Nair et al. [30] as a call with a rich harmonic structure, a mean F0 of around 680 Hz and a mean call duration of approximately one second in adult females; these trumpets were recorded in the contexts of disturbance (by vehicles, humans or other species), play, inter-specific aggression and while running out of a waterhole. De Silva [16] reported trumpets to show low harmonicity and a mean F0 of around 540 Hz, but also around one second long–uttered mainly in the context of aggression, fear, excitement and disturbance. While the general acoustic structure of the trumpet is described and the broad context is known to be arousing situations triggered either by conspecifics or external stimuli, little is known on the information content and whether trumpets as arousal calls encode information about the caller’s phenotype or identity.

Investigating the information content of the different vocalizations within a species’ vocal repertoire is essential to more holistically understand its communication system. Vocalizations of various non-human mammalian species have been shown to carry information regarding phenotype [e.g. 31, 32, review 33], sex [e.g. 31, 32, 34], context [e.g. 35, 36], caller identity [e.g. 32, 34, 35, 37–42] and emotional state of the caller [e.g. 39, 43, 44]. Caller identity is specifically relevant in social species, but has been found in most mammal and bird species investigated so far. Vocal identity is partly related to individual anatomical and morphological differences in the sound producing structures (the source or other vocal tract structures), as well as in the physiology of sound production and internal factors (reviewed in [33]).

So far it has been shown that elephant rumbles convey much information in African savanna elephants [23, 24, 45–49]. Less research has so far addressed rumbles in Asian elephants, but they are also individually distinctive and vary with contextual arousal [16, 29]. Besides rumbles, our knowledge about information content in elephant vocalizations is limited. Sharma et al. [29] reported variation in the information content of Asian elephant trumpets in relation to context: trumpets were significantly shorter when emitted in a disturbed context (induced by humans or other species) compared to an undisturbed state (contact calls or during social interaction). That study, however, recorded wild elephants in dense habitat, and observers were unable to identify the calling individual. Analyses on information content about individual distinctiveness are particularly difficult to achieve in call types that are predominantly produced in high arousal contexts, since they require a certain amount of vocalizations of known individuals; and the trumpets of neither elephant species have been tested for individual distinctiveness so far. Importantly, however, squeaks of Asian elephants (a call type unique to Asian elephants and also produced in situations of arousal and conflict) have recently been shown to be individually distinctive [50]. Accordingly, individuality is conveyed in call types other than rumbles in Asian elephants. Signaling individuality–particularly when aroused–is no doubt crucial for social animals such as elephants that rely on both the physical and emotional support of affiliates. The roars of infant African elephants, which are also uttered in a state of arousal, however, have a rather low potential to encode information on the caller’s identity, but do contain information about the emotional state of the calf. Roars uttered in higher urgency were longer in duration and had the lowest harmonics-to-noise ratio, thus containing chaos [43].

Non-linear phenomena (NLP) typically occur in self-oscillating systems when driven to the limit or where multiple oscillators interact [51]. They are an integral part of mammal vocalizations and have indeed been suggested to facilitate individual recognition [52, 53]. While some acoustic cues (e.g. F0) will vary along a continuum, the principles of non-linear dynamics predict another source of variability, leading to qualitative variation in a call. A certain call may occupy a stable oscillation regime for one individual, while the same call may cross a transition point to an unstable regime in another individual [51]. Individual differences in anatomy or neural control, combined with non-linearities in the production system, can lead to pronounced differences in call morphology between individuals [51]. Further, NLP supposedly facilitate unpredictability, which makes them more difficult to habituate to and ignore [54–56]. In non-human mammals NLP occur, for example, in the ultrasonic vocalizations of rodents [e.g. 57–60], but have also been reported in the whistle vocalizations of camels [61], the bugles of North American wapitis *Cervus canadensis* [62], in African wild dogs *Lycaon pictus* [63], dog-wolf mixes [64], domestic dogs *Canis lupus forma domestica*, piglets *Sus scrofa forma domestica*, Japanese macaques *Macaca fuscata* [65], rhesus macaques *Macaca mulatta* [51], common chimpanzees *Pan troglodytes* [66] and West Indian manatees *Trichechus manatus* [67].

A system that is vibrating in phase (this can be the vocal folds, but also other structures that vibrate) usually generates a highly tonal sound made up of a F0 and its harmonics [68]. NLP occur when the system gets somehow out of phase [69, 70], when the vibrating structures desynchronize in the horizontal and vertical plane [63, 71]. In sounds produced by the vocal folds, ‘subharmonics’ occur when one vocal fold vibrates at exactly two or three times the frequency of the other, which results in parallel bands between the preexisting harmonics (period doubling) or at the multiples of one third of the original pitch (period tripling). Two vocal folds vibrating independently generate two independent fundamental frequencies (F0 and G0), a phenomenon known as ‘biphonation’. Linear combinations of these two fundamentals can also lead to additional bands in the spectrogram (‘frequency bands’), above and below the fundamentals. Non-periodic irregular vibration of the vocal folds leads to ‘deterministic chaos’ (henceforth referred to as chaos), which is characterized by a broadband spectrum with energy at many frequencies. Several of these non-linear vibratory regimes often occur within one vocalization, and transitions between them are termed ‘bifurcations’. Moreover, the onset from resting structures to oscillation or the sudden transition from one F0 to another, a ‘frequency jump’, is also considered a ‘bifurcation’ [reviewed in 51, 63, 72]. Another phenomenon that can appear in the spectrogram are ‘sidebands’, which occur when a carrier frequency is modulated with an additional (second) low F0, which results in amplitude modulation of the carrier frequency (period of modulation is equal to the period of the low frequency). They appear as additional bands above and below the carrier frequency [reviewed in 73, 74].

In elephants, NLP have been documented in adult African elephant rumbles [45], infant African elephant roars [43], in trumpets, rumbles and roars of Asian elephant calves [17] and in squeaks of Asian elephant calves [17, 50] and adults [50]. In infant African elephant roars, the observed NLP increase with increasing level of arousal, probably indicating a high level of urgency [43]. Furthermore, NLP in infant elephant vocalizations might function to increase unpredictability, making the signals harder to ignore by mothers, allomothers and other group members [17, 43]. NLP in the trumpets of adult Asian elephants may serve a similar purpose.

Very little is known about sound production mechanisms in elephants. Rumbles in African elephants are most likely produced via passive vocal fold vibration [21]. The vocal folds of elephants are massive (10 cm in a 25-year-old female), and it is suggested that trumpets are produced via a different production mode. Just recently, Beeck et al. [50] found evidence that the high-frequency squeaks of Asian elephants are produced via lip buzzing by forcing air from the oral cavity through the tensed lips, inducing self-sustained lip vibration. The high F0 in trumpets also suggests that they may be generated with a secondary source other than the vocal folds [12, 13]. Boas & Paulli [75] proposed that the margins of the rigid cartilaginous plates on the lateral sides of the nasal cavities are set into vibration by vigorously exhaling air through the trunk. Generation via the trunk, an enormously flexibly muscular hydrostat with over 100,000 muscle fiber bundles [76], might enable a specific plasticity for sound production. Since aroused elephants (whether in a positive or negative context) are often in motion, another source of variability is respiration and general body posture and movement beyond the position, tension, and extension of the trunk; e.g. running might lead to a specific pulsated structure like in the ‘pulsated-play-trumpets’ described by Poole [14] in African elephants.

Here we investigate the acoustic structure of the elephant trumpet, a call type strictly associated with high excitement, distress and arousal, and investigate its potential for coding individuality. We also address NLP in trumpets in order to discuss their possible function in calls of adult individuals. This provides new insights into the adaptive function of this characteristic vocalization type within the Asian elephants’ communication system beyond signaling arousal and excitement.

## Methods

### Study subjects and housing

The subjects in this study were 12 female Asian elephants aged between 11 and 60 years with a shoulder height ranging from 2.11 m to 2.55 m (Table 1). The elephants were kept in chain-free corrals of around 0.25 ha to 1.55 ha in size, in groups of two to three individuals at Tiger Tops Tharu Lodge (a private facility) near Chitwan national park, Nepal. One individual was housed alone but had visual and acoustic contact to her conspecifics. The animals were taken for daily walks by their mahouts (elephant caretakers) and often allowed to freely interact socially in bigger groups at certain sites for bathing and browsing. Bathing usually took place at the nearby river Narayani. Before and/or after bathing the elephants were browsing at an open grassland area next to the river, but sometimes were also allowed to freely browse through the forest in between walks. All elephants were habituated to the presence of humans.

**Table 1:** Study subjects and collected data.

*Individual*	*Age*^*1*^ *(y)*	*Shoulder height*^*2*^ *(m)*	*N trumpets analyzed* ^*3*^
Chan Chun	45	2.47	25
Dhibya	48	2.50	22
Dipendra	60	2.43	20
Saraswati	27	2.40	40
Sona	40	2.54	30
Sunder	46	2.41	69
Champa*	41	2.40	3
Kanchi*	11	2.11	3
Hira*	45	2.55	1
Pawan*	55	2.41	1
Sita*	46	2.34	0
Raj*	42	2.49	0

^1^Age of the elephant according to their mahout at the time of recording.

^2^Mean value of two measurements.

^3^Number of trumpets of which the acoustic parameters of F0 could be extracted.

*Individuals excluded from analysis due to low sample size.

### Recording context

The trumpets for this study were recorded in two different contexts–greeting and disturbance. To induce the greeting context, we conducted social separation experiments during which the focal individual stayed at the corral while the other elephant housed with the focal was lured away with food by the mahout to a spot out of sight for the focal individual (approximate distance 100–200 m). The elephant that left was allowed to return at any time or was brought back after a maximum of 40 min separation if she did not return by herself. Regarding one group composed of three individuals, two elephants were brought to two separate spots not visible to the focal individual. Animals from neighboring corrals were also brought out of sight of the focal before the start of the experiment.

A greeting ceremony usually involved the incoming elephant(s) running towards the focal individual while vocalizing. Once reunited the elephants would stay close to each other, touch each other with their bodies and trunks, and often also urinate [77, 78]. One individual (Sunder) had an impaired hind leg due to a previous injury and stayed behind at the corral when the other two elephants she was housed with were taken out for walks. Their return often resulted in a greeting ceremony, and trumpets from these greetings were also included into our analysis.

The other context category included trumpets in response to disturbance through dogs, cars or commands (when the mahout told them to lie down or walk, etc.). Behavior in response to disturbance usually involved an upright body posture, high-held head and spread ears or rapid ear flapping, typically also accompanied by trunk bounces. In the case of disturbance by dogs, the elephants would also turn towards them and sometimes (mock-)charge them, i.e. rapidly approaching the dog either with (real charge) or without (mock-charge) attempt to make contact with the target (for a detailed ethogram on elephant behavior see [77]).

### Ethical statement

This research was carried out under approval from the animal ethics and experimentation board of the faculty of life sciences from the University of Vienna (No.2018-001). All animal owners consented to the conducted experiments, the data collection, and publication of the data (written and verbally).

### Data collection

Two observers recorded simultaneously with two recorders at different areas within the field site during daytime between 6 a.m. and 8 p.m. from February to April 2018, and one person also did recordings at the same field site in October 2018. This yielded a total of 58 days and 548 hours of acoustic recordings. Recording sessions were done at different areas, inside and directly outside the corrals and at bathing and browsing sites. We recorded the elephants’ vocalizations at a distance ranging from 3–70 m. For each recorded vocalization the identity of the caller, the context, behavior and trigger as well as the location and approximate recording distance were noted. We determined the caller ID through directional hearing along with observed behavior. During the conducted social separation experiments the observers were recording and videotaping. Greetings were often accompanied by overlapping calls and many consecutive vocalizations in a short time, while the elephants were also moving (walking, running, turning) a lot. The use of two cameras and sound recorders facilitated following the elephants as they were moving.

Acoustic data were obtained using two omni-directional Neumann microphones (KM 183) modified for recording frequencies below 20 Hz (flat recording down to 5 Hz) connected to a 722 Sound Device HDD recorder and a 633 Sound Device, respectively, at 48 kHz sampling rate (recording response of the system down to 10 Hz). For video recordings we used a Camcorder Panasonic Sowe and a Sony Camera FD53.

### Acoustic analysis

**Fig 1:**
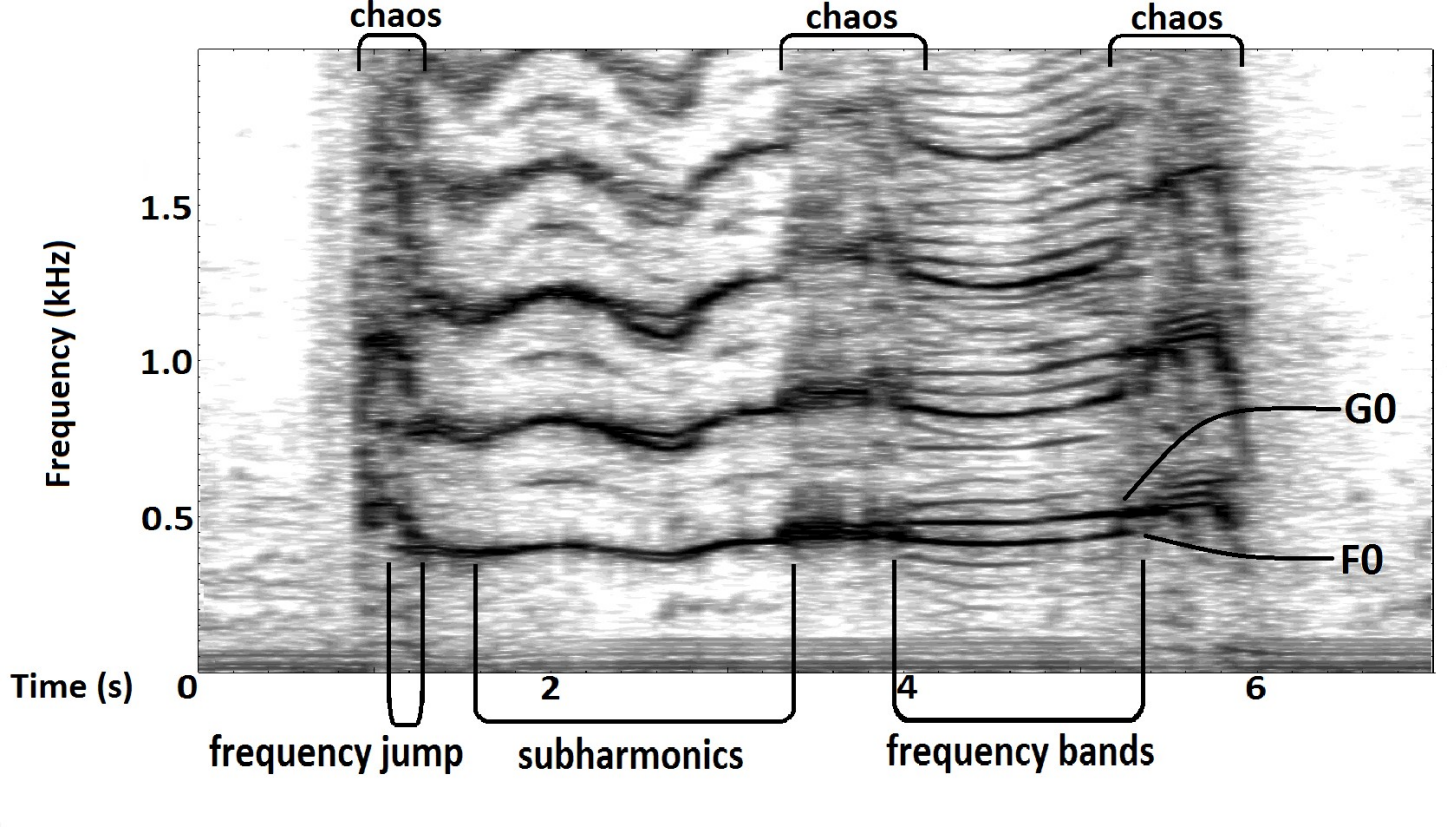
Spectrogram of a trumpet showing all different types of NLP.

We performed acoustic data annotation using the acoustic analysis tool STx 4.4.10 from the Acoustic Research Institute at the Austrian Academy of Science [79]. Trumpets were identified based on field notes, by listening and visual examination of the spectrograms. For trumpets in the context of greeting in the course of the social separation experiments, we also reviewed video footage to identify the calling individual. The identified calls were then tagged and the respective annotations added. We used a customized template to annotate calls (e.g. the vocalizing individual, context, behavior, location). For each trumpet, the appearance of NLP–i.e. biphonation, subharmonics, frequency jumps and chaos–were noted (Fig 1). We distinguished between subharmonics and other phenomena like frequency bands or sidebands by manually measuring the spacing between the bands in the spectrogram. The occurrence of the different NLP was then calculated as a percentage of all analyzed trumpets and all trumpets per individual.

To extract source-related acoustic features, we used a customized semi-automatic analysis tool in Matlab [80]. The tool computes Fast Fourier spectrograms (frame size: 100 ms; step size: 10 ms) of the input calls, and the contours of F0 were then traced manually. In case of biphonic trumpets, we traced the lower frequency component as F0, and the higher frequency component as G0. From the traced contours, we automatically extracted and calculated frequency-, contour-, shape- and temporal-related parameters (Table 2).

**Table 2:** Description of the acoustic parameters measured [23, 81].

Acoustic parameter	Description
***Absolute frequency parameter (Hz)***	
Start, Mid, Finish Frequency	Frequency at the temporal start, middle and end of the fundamental frequency
Minimum and Maximum Frequency	Lowest and highest measured frequency of the fundamental
Mean Frequency	Calculated as average frequency across the fundamental
Mean 1^st^, 2^nd^ and 3^rd^ Third	Mean fundamental frequencies of first, second and third part of the sound segment
Median Frequency	Median value of the fundamental frequency
Frequency Range *	Calculated as maximum frequency minus minimum frequency
***Temporal parameters***	
Duration	Temporal distance of trumpet measured in seconds
Minimum * and Maximum Frequency Location	Location of the minimum and maximum frequency on the contour, given as percentage of duration
TimeMin/Max *	Temporal distance between minimum and maximum frequency
***Shape and contour parameters***	
COFM—Coefficient of frequency modulation [82]	Calculated variable that represents the amount and magnitude of frequency modulation across the trumpet, computed by summing the absolute values of the difference between sequential frequencies divided by 10,000
Jitter Factor [83]	Calculated variable that represents a weighted measure of the amount of frequency modulation, by calculating the sum of the absolute value of the difference between two sequential frequencies divided by the mean frequency. The sum result is then divided by the total number of points measured minus 1, and the final value is obtained by multiplying it by 100
Frequency Variability Index [83]	Calculated variable that represents the magnitude of frequency modulation across a contour, computed by dividing the variance in frequency by the square of the average frequency of the contour and then multiplying the value by 10
Inflection Factor	Percentage of points showing a reversal in slope
Start, Middle and Final Slope	Calculated as (Frequency 20-Frequency 1)/(Time 20-Time 1)(Frequency 40-Frequency 20)/(Time 40-Time 20) (Frequency60-Frequency 40)/(Time 60-Time 40)

* Variables not included into statistical analysis.

### Statistical analysis

Our data set for statistical analysis consisted of 120 trumpets from 6 elephants (20 calls per individual). For individuals from whom we had more than 20 calls, we included 20 trumpets into analysis, while keeping the data set as balanced as possible regarding the two categories of context, greeting and disturbance (Table 3). When we had less than 10 trumpets per context and individual, from which we were able to extract the acoustic parameters of F0 from, we prioritized those. We then randomly chose trumpets in the other context to add-up to 20 calls per individual. We log_10_ transformed the following acoustic parameters of the F0: COFM, Jitter Factor, Frequency Variability Index, Finish Frequency, Minimum Frequency, Maximum Frequency, Mean Frequency, Duration, Start Slope and Mean 3^rd^ Third to approximate normal distribution. As the parameter Start Slope consists of positive and negative values, we added the minimum plus one before transformation. The parameter Minimum Frequency Location was excluded from statistical analysis due to its strong bimodal data distribution. Since all individuals included in our analysis were adult females and the maximum difference in mean shoulder height between individuals was only 14 cm (Table 1), we did not include shoulder height as a factor in the statistical analysis.

**Table 3:** Number of trumpets per individual and context included into statistical analysis.

	*Context*	
*Individual*	Greeting	Disturbance
Chan Chun	10	10
Dhibya	7	13
Dipendra	9	11
Saraswati	11	9
Sona	4	16
Sunder	12	8

To test whether trumpets are individually distinctive and structurally context dependent, we first performed a varimax rotated principal component analysis (PCA) with Kaiser-normalization for data reduction–after testing the data for suitability using the Kaiser-Meyer-Olkin (KMO) measure of sampling adequacy and performing a Bartlett’s test of Sphericity. Factors with an eigenvalue above one were retained and used for classification analysis.

To test the classification of trumpets based on individuality and context, we performed discriminant function analysis (DFA) with the obtained factors from the conducted PCA. To confirm that classification results do in fact derive from differences among individuals and are not context related, or contrariwise, we also conducted a permuted discriminant function analysis (pDFA), used for non-independent two-factorial data sets when one factor in nested in another [84]. In order to control for non-independence of the data, the permutation operates on units defined by the combination of factors (in this study individuals and context). The pDFA function balances the sample size to derive the discriminant functions based on the smallest sample of combination of factors and uses the remaining cases as test set. For our data set that means the restricting combination of factors are trumpets from Sona in the context of greeting, resulting in four calls per individual and context (Table 3). For the first pDFA we determined individuality as the test factor and context as the control factor, vice versa for the second pDFA; we used 100 random selections and 1000 permutations for each test. The pDFA was performed using a function (provided by R. Mundry) based on Ida of the R package MASS [85]. G0 data in biphonic calls were not sufficient to perform comparable statistical analysis. The PCA and DFA were conducted with SPSS software version 23 and for the pDFA we used RStudio v.1.1.463 (R 3.6.1).

## Results

### Acoustic structure

Trumpets showed a mean F0 of 474 ± 70 Hz and a duration of 1.38 ± 1.46 s (N_indiv._ = 6, N_calls_ = 206). In biphonic calls, the mean G0 was 508 ± 69 Hz (N_indiv._ = 6, N_calls_ = 117). Individual’s mean F0 ranged from 377 ± 33 Hz (Chan Chun) to 528 ± 41 Hz (Saraswati). Regarding biphonic calls, the individual’s mean G0 ranged from 431 ± 59 Hz (Chan Chun) to 562 ± 38 Hz (Sunder), so the lowest mean F0 and mean G0 were found in the same individual (Table 4).

**Table 4:** Acoustic parameters measured in female Asian elephant trumpet calls presented as mean ± SD.

*Individual*	*All*	*Chan Chun*	*Dhibya*	*Dipendra*	*Saraswati*	*Sona*	*Sunder*
**N F0**	206	25	22	20	40	30	69
***Mean F0 (Hz)***	474 ± 70	377 ± 33	417 ± 34	402 ± 52	528 ± 41	463 ± 40	522 ± 38
***Min F0 (Hz)***	409 ± 64	337 ± 25	380 ± 26	343 ± 53	434 ± 42	373 ± 45	465 ± 41
***Max F0 (Hz)***	514 ± 75	426 ± 48	450 ± 35	432 ± 54	572 ± 50	513 ± 46	559 ± 48
***Range F0 (Hz)***	105 ± 54	89 ± 44	70 ± 19	89 ± 38	138 ± 59	140 ± 54	94 ± 50
***Duration F0 (s)***	1.38 ± 1.46	3.43 ± 2.33	3.11 ± 1.71	0.56 ± 0.23	0.97 ± 0.36	0.38 ± 0.11	1.01 ± 0.39
**N G0**	117	20	21	11	7	11	47
***Mean G0 (Hz)***	508 ± 69	431 ± 59	455 ± 23	494 ± 64	548 ± 58	505 ± 43	563 ± 38
***Min G0 (Hz)***	458 ± 73	380 ± 44	423 ± 24	452 ± 71	465 ± 40	416 ± 84	516 ± 48
***Max G0 (Hz)***	545 ± 70	479 ± 64	496 ± 36	521 ± 70	581 ± 70	549 ± 38	596 ± 47
***Range G0 (Hz)***	88 ± 53	99 ± 45	73 ± 35	69 ± 37	116 ± 69	133 ± 86	79 ± 46
***Duration G0 (s)***	1.72 ± 1.71	3.71 ± 2.13	3.06 ± 1.75	0.54 ± 0.26	1.26 ± 0.39	0.35 ± 0.09	0.94 ± 0.35

**Fig 2:**
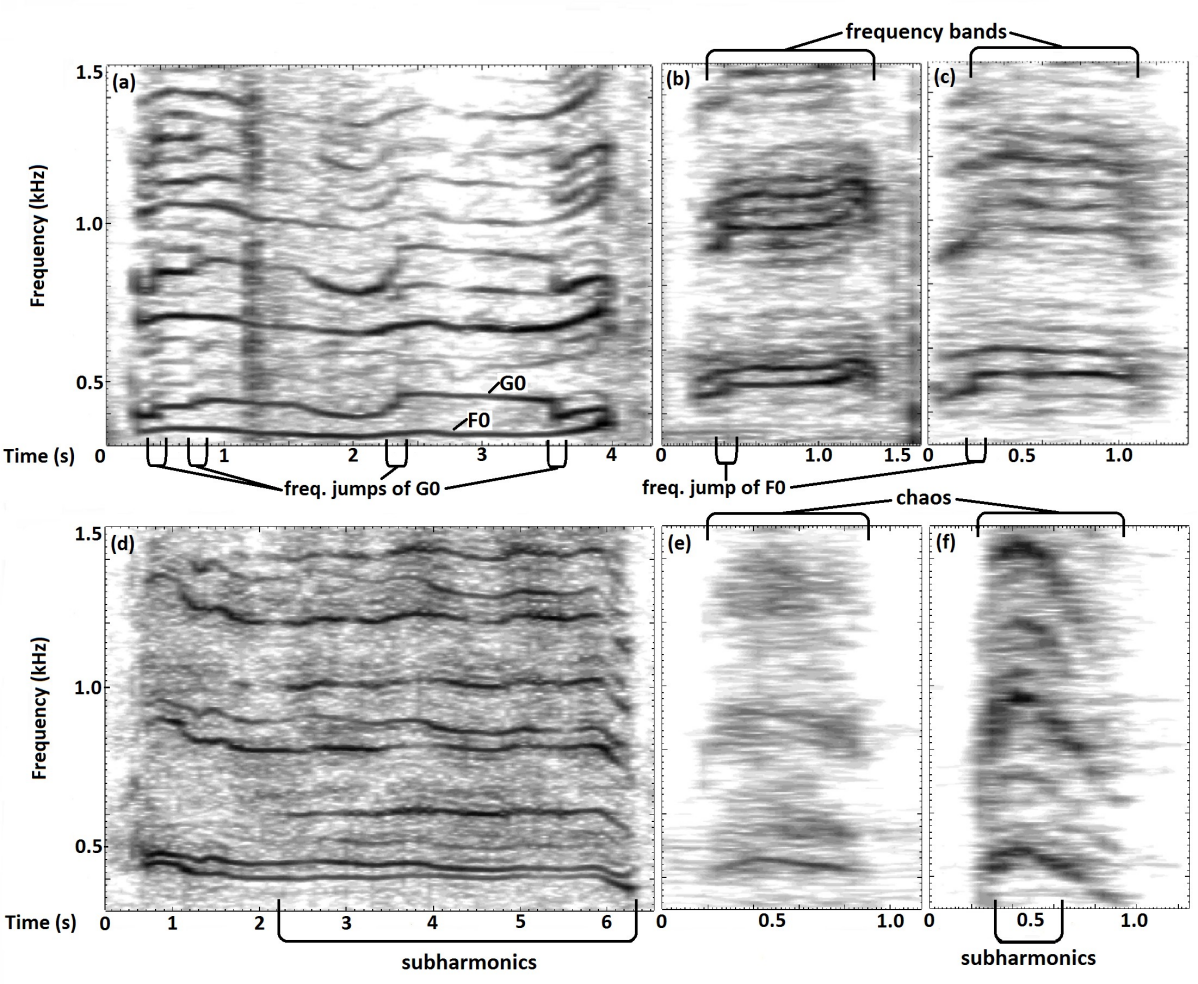
Spectrographic representations showing the acoustic variation and different types of NLP in trumpets of different individuals. (a) Chan Chun–S1 Audio, (b) Sunder, (c) Saraswati, (d) Dhibya, (e) Dipendra, (f) Sona–S2 Audio.

The trumpets of two individuals had a mean duration of over three seconds (Chan Chun: 3.43 ± 2.33 s (Fig 2A), Dhibya: 3.11 ± 1.71 s (Fig 2D)); in comparison, the other four individuals showed a mean duration of only up to one second (0.38 ± 0.11 s to 1.01 ± 0.39 s). All values are given as mean ± SD, as they are presented in Table 4. Mean values ± SD for all extracted parameters described in Table 2 are presented in S1 Table (F0) and S2 Table (G0).

### Classification based on individuality and context

The PCA was justified by the KMO criterion (0.831) and Barlett’s test of Sphericity (*X*^*2*^ = 4,549.843, *df* = 171, *p* = < 0.001) and reduced 20 acoustic parameters to four principal components (PC) explaining 86.1% of the total variance. Absolute frequency parameters were assigned to PC 1 explaining 44.3% of the variance. Shape- and contour-related variables correlated with PC 3 (13.7%), whereas PC 2 (17.7%) and PC 4 (10.5%) correlated with both shape- and contour-related variables as well as temporal parameters. Loading values for each parameter are shown in Table 5.

**Table 5:** Results of the PCA. Conducted with varimax rotation and Kaiser normalization performed on a set of 120 trumpets from 6 individuals (20 per individual).

*Parameter F0*	*PC*			
	1	2	3	4
log_10_ Mean Frequency	0.981	-0.150	0.081	-0.014
Median Frequency	0.961	-0.219	0.110	0.001
log_10_ Mean 3^rd^ Third	0.957	0.078	0.004	0.240
Mean 2^nd^ Third	0.937	-0.271	0.145	0.070
log_10_ Maximum Frequency	0.933	-0.127	0.300	-0.073
Mid Frequency	0.927	-0.292	0.152	0.061
Mean 1^st^ Third	0.905	-0.192	0.091	-0.338
log_10_ Minimum Frequency	0.903	0.134	-0.320	-0.083
log_10_ Finish Frequency	0.860	0.233	-0.185	0.283
Start Frequency	0.739	0.037	-0.062	-0.585
log_10_ Duration	-0.227	0.855	0.290	-0.154
Final Slope	-0.082	0.822	-0.275	0.188
Inflection Factor	-0.063	0.685	-0.133	0.159
log_10_ Start SlopeP1	0.061	-0.550	0.216	0.371
log_10_ COFM	0.123	0.093	0.972	0.026
log_10_ Frequency Variability Index	0.028	-0.464	0.833	0.009
log_10_ Jitter Factor	0.064	-0.601	0.647	0.200
Peak Frequency Location	0.064	-0.139	0.087	0.802
Middle Slope	0.011	0.467	-0.058	0.683
Eigenvalues	8.419	3.364	2.593	1.989
Percentage of variance	44.312	17.706	13.650	10.468
Percentage of cumulative variance	44.312	62.018	75.667	86.135

The DFA testing for individuality showed 71.7% correct classification applying cross-validation (N_cases_ = 120). The pDFA testing for individuality, controlling for an effect of context, resulted in 71.5% correctly cross-validated classifications (*p* = 0.003). This shows that individuals can be discriminated based on acoustic features of the F0 of their trumpet vocalization.

The DFA testing for context showed 58.3% correctly cross-validated classifications (N_cases_ = 120). The second pDFA testing for context with individuality as a control factor resulted in 55.6% correctly cross-validated classifications (*p* = 0.532). This indicated no effect of the two categories of context on the acoustic features of F0.

### Non-linear phenomena

NLP were observed in all trumpets, with chaos being the most common type, even occurring in every recorded call in two out of the six individuals. The least common type was subharmonics, which were present in only 26% of all analyzed trumpets, but particularly high in one individual with 86%. The smallest percentage of subharmonics relative to entire call duration registered by us was 13%. In the second least common type of NLP, the frequency jumps, the percentage was notably high in one individual. The smallest registered and counted frequency jump in this study was 15 Hz. The two individuals that showed the longest call duration (Table 4) also showed the highest percentage of biphonic calls as well as the fewest occurrences of chaos in trumpets. The smallest percentage of biphonation over whole call duration we found was 14%. Additional frequency bands occurred in 55% of trumpets and appeared between 30 and 70 Hz above and below the carrier frequencies, F0 and G0 respectively. Percentages of trumpets with the different kinds of NLP in all analyzed trumpets and in each individual are given in Table 6; Fig 2 illustrates the occurrence of different NLP in different individuals.

**Table 6:** Occurrence of NLP as a percentage of all trumpets per individual.

*Individual*	*all*	*Chan Chun*	*Dhibya*	*Dipendra*	*Saraswati*	*Sona*	*Sunder*
N	206	25	22	20	40	30	69
***Biphonation***	59%	80%	95%	55%	20%	40%	71%
***Subharmonics***	26%	44%	86%	10%	13%	13%	19%
***Frequency jump***	52%	36%	32%	30%	80%	57%	54%
***Chaos***	80%	16%	32%	100%	90%	100%	99%

## Discussion

Our study shows that trumpets of adult female Asian elephants can be individually distinguished based on the acoustic features of the F0 contour and call duration. Since our results for the DFA and pDFA (controlling for behavioral context) barely differ (by only 0.2% correctly cross-validated classifications) we conclude that the behavioral context considered in this study had no effect on individual trumpet distinctiveness. Furthermore, this indicates that acoustic cues are individually distinctive enough to provide similar results even with the considerably smaller sample size used to calculate the discriminant function in the pDFA compared to the DFA. The Asian elephant is a highly social species that lives in matriarchal family groups [5, 6], forms close social bonds with unrelated individuals in captivity [86–88], and even reassures conspecifics in distress [89]. Thus information about caller identity is certainly valuable, for example to facilitate social interactions or to specifically evoke supportive behavior of bond partners. This is especially valid for a call that, as we found corresponding with previous studies, is associated with excitement, arousal, disturbance and distress [16, 29, 30]. In the future, this calls for playback experiments to clarify if and how well Asian elephants are able to recognize and distinguish familiar conspecifics based on their trumpet vocalization. NLP may enhance individual acoustic distinctiveness in mammals [52, 53, 62]. Our results indicate that this might be the case in the trumpets of adult Asian elephants, since the occurrence of the NLP types varies clearly between the individuals in this study (Table 6).

We found no statistical evidence for a difference in temporal and acoustic parameters of F0 between the greeting and disturbance context. Sharma et al. [29] also found no influence of context on frequency-related parameters (mean F0, mean F0 range, mean Formant 1, mean Formant 2) in trumpets of wild Asian elephants, but did report that trumpets were shorter in ‘disturbed’ versus ‘undisturbed’ behavioral contexts. Even though their context category ‘disturbed’ may be similar to the context disturbance in our study, their context ‘undisturbed’ included male-female, female-female and mother-calf interactions, along with intra- and intergroup antagonistic interactions, which is not comparable to our explicit context of greeting solely among adult females of the same social group. The observed difference in duration in Sharma et al.’s [29] study may therefore derive from contexts we did not include in our analysis. Overall, the contexts under which trumpets occurred in our study were limited. We, for example, did not record trumpets during intra-specific aggression. The study elephants had well-established bonds and their mahouts avoided socializing specific individuals to prevent any type of aggressive behavior. We also observed little play behavior and did not record any trumpets in this context either. The youngest individual included into statistical analysis was 27 years old, while the other five were between 40 and 60 years. In contrast to adults, Asian elephant calves become increasingly excited when playing and trumpet predominately during play behavior [17]. Based on their recording conditions of Asian elephants in the wild, Sharma et al. [29] were not always able to identify the vocalizing individual, and the effect of individual differences on their results cannot be excluded. Poole [14] postulated for African elephants that during social events (e.g. mating, greeting, conflict) rumbles may define context, whereas the trumpet vocalization may serve to express the level of excitement and to emphasize the importance of an event. The same could be true in Asian elephants.

The most common NLP in our study was chaos. NLP, and specifically chaotic calls, are harder to ignore and take longer to habituate to in the alarm calls of meerkats *Suricatta suricatta* [55] and yellow-bellied marmots (*Marmota flaviventris*) [56]. It makes sense for a call such as the elephant trumpet, which is mostly uttered in a state of arousal and in response to disturbance [16, 30], to exhibit a high rate of chaos. This might make it more likely to evoke a response from conspecifics. Also, in infant African elephant roars [43], infant giant panda *Ailuropoda melanoleuca* vocalizations [44], and calls in various primate species [90–93], the amount of NLP increased with the level of the caller’s arousal. If the same is true in elephant trumpets, then the level of chaos (possibly along with other NLP or parameters) may also convey the level of excitement in social events, as Poole [14] suggested. This, however, remains to be confirmed in any elephant species. Such a hypothesis would need to be tested by a detailed record of behavior and physiological measurements to determine arousal level along with sound recording. This is highly challenging even in captive settings.

Trumpets showed a mean F0 of 474 ± 70 Hz and a duration of 1.38 ± 1.46 s (N_indiv._ = 6, N_calls_ = 206, Table 4). This duration is longer than previously reported [16, 29, 30], which may reflect the fact that two out of the six individuals whose trumpets we analyzed had a remarkably long mean duration of over three seconds (Table 4), with the longest value being 8.04 s, uttered by Chan Chun. Mean trumpet duration in African elephants has been reported from 0.7 up to 5 s [10, 11, 14], to which our findings correspond better than to the previous results in Asian elephants. Our mean F0 of 474 ± 70 Hz (Table 4) is slightly lower than that of de Silva [16] of 542 ± 27 Hz, but clearly lower than the one reported by Sharma et al. [29] of around 600 Hz. This may be because both of the latter studies defined adult females as being over 10 years of age, while female Asian elephants continuously grow until an age of 15 years and rapidly gain weight until an age of 19 years [94]. Considering that the youngest individual in our study was 27 years old, the difference in F0 might therefore derive from a difference in size of the recorded individuals. Nair et al. [30] did differentiate between age classes and reported higher frequencies in the trumpets of young female Asian elephants (juveniles and calves; 787 ± 49 Hz) versus adult females (adults and sub-adults; 678 ± 29 Hz). Compared to the African savanna elephant trumpet, with a mean F0 of 300–390 Hz [10, 11, 14], Asian elephant trumpets seem to be generally higher in frequency. Since the production mechanism has not yet been determined, this observation warrants further research, i.e. comparing individuals of the same age, size and sex of both species. The reports of higher frequencies in Asian elephant calves [17] and in younger and smaller females [30] indicate that the source of trumpet production varies with caller size. Contrary to this, Nair et al. [30] observed the highest F0 (>800 Hz) in trumpets of adult males, the age group with the biggest body size.

Our results support previous observations that NLP are very common in Asian elephant trumpets [17]. We were able to observe all NLP types that typically occur in laryngeal vocalizations of other species [51, 63, 72]. This strongly suggests two simultaneously vibrating structures, especially because 59% of all analyzed trumpets displayed biphonation. The vocal folds of elephants are suggested to be too massive [21] to be the source of the high-frequency trumpet [12, 13]. We support the hypothesis of a secondary source other than the vocal folds [12, 13], for example structures or tissues that are vibrating during strong exhalation through the trunk, supposedly at the base of the trunk. Chaos, often an indicator of increased vocal effort, is associated with increased air pressure and tension of the vocal folds, which leads to irregular vibration [51, 63, 95, 96]. Since chaos is also observed in trumpets, a behavior similar to that of the vocal folds is to be expected for the vibrating sound source in trumpets. The origin of the occurring frequency bands can be explained by an interaction between the two different fundamentals (F0 and G0) [62, 63].

Concerning the observed individual differences in NLP, note that Chan Chun and Dhibya–the two individuals that showed the least occurrence of chaos–also displayed the highest abundance of biphonation and subharmonics (Table 6) as well as the longest mean call duration (Table 4). Our sample size is too small to draw general conclusions about these coherences. Possibly, variation in NLP derives from individual anatomical differences. Elephants are capable of vocal imitation [97, 98] and Stoeger et al. [12] suggested that elephants have to learn and practice how to trumpet after observing infant African elephants. Elephants might be able to intentionally vary the trumpet vocalization by modifying air speed, trunk shape, body posture and muscle movements while trumpeting [14]. The fact that the trumpets of four individuals contained noticeably more chaos than the trumpets of the other two (Table 6) may also reflect different levels of arousal of the vocalizing individuals since chaos has been shown to increase with arousal [43, 44, 90–93]. As recording context did not differ, this might reflect a difference in personality. The shorter mean call duration in the same individuals (Table 4) would then support Sharma et al.’s [29] findings of trumpets being shorter in ‘disturbed’ context. Since we did not find any significant differences regarding context, we suggest that these findings may also derive from individual differences.

In total, we recorded vocalizations from 12 individuals but could not record any trumpets from two individuals. Some elephants were more likely to trumpet than others. Here, again, individual differences in terms of personality or individual experience and history might explain the dissimilarities in vocal behavior. Trumpet development and ontogeny needs to be investigated in more detail, along with addressing the questions whether NLP reflect arousal levels, and whether the acoustic structure is influenced by vocal learning processes (e.g. imitation and call convergence) among individuals (of varying age groups and sex) of social groups and affiliates.

### Conclusion

We report that elephant trumpets are individually distinctive based on the parameters of their fundamental frequency, and non-linear phenomena might even enhance distinctiveness. Our results further indicate that trumpets might not be strictly context-specific. Further investigations should examine trumpets of males and include a detailed record of behavior and physiological stress analyses to determine arousal levels. Playback experiments are necessary to reveal the adaptive function of this pronounced and important vocalization type.

## Supporting information

S1 Table Mean ± SD for all extracted acoustic parameters of the fundamental frequency (of the lower frequency component for biphonic trumpets). (PDF)

S2 Table Mean ± SD for all extracted acoustic parameters of the fundamental frequency of the higher frequency component of biphonic trumpets. (PDF)

S3 Table Dataset used for descriptive statistics. (XLSX)

S4 Table Dataset used for statistical analysis. (XLSX)

S1 Audio Long biphonic trumpet. Uttered by Chan Chun; respective spectrogram in Fig 2A in the article.
(WAV)

S2 Audio Short chaotic trumpet. Uttered by Sona; respective spectrogram in Fig 2F in the article.
(WAV)
