# Supplementary material for: Acoustic structure and information content of trumpets in female Asian elephants (Elephas maximus)
Source: PLoS One. 2021 Nov 23;16(11):e0260284. doi: 10.1371/journal.pone.0260284 (PMC8610244; doi:10.1371/journal.pone.0260284)
Supplement: S1 Table — (PDF) [file pone.0260284.s001.pdf]

S1 Table: Mean ± SD for all extracted acoustic parameters of the fundamental frequency (of the lower frequency component for biphonic trumpets).

F0	all (206)	Chan Chun (25)	Dhibya (22)	Dipendra (20)	Saraswati (40)	Sona (30)	Sunder (69)
**Absolute frequency parameters (Hz)**							
Start F0	443.45 ± 64.55	386.72 ± 45.59	433.35 ± 40.51	350.72 ± 51.26	449.99 ± 42.40	455.24 ± 48.44	485.19 ± 54.37
Mid F0	490.69 ± 77.76	372.11 ± 43.78	422.42 ± 35.72	425.90 ± 50.31	556.97 ± 50.65	497.35 ± 42.99	532.88 ± 43.23
Finish F0	433.98 ± 72.74	360.40 ± 37.43	383.75 ± 22.64	383.81 ± 62.98	481.80 ± 58.49	374.07 ± 45.72	489.53 ± 41.59
Minimum F0	409.09 ± 63.96	337.05 ± 24.66	380.03 ± 25.65	343.40 ± 52.62	433.82 ± 41.85	372.99 ± 45.40	464.86 ± 41.42
Maximum F0	514.45 ± 74.75	425.77 ± 47.97	449.55 ± 34.91	432.13 ± 54.07	571.72 ± 50.49	513.02 ± 46.18	558.55 ± 48.12
Mean F0	474.21 ± 69.89	376.50 ± 33.29	417.45 ± 33.62	401.60 ± 51.51	527.71 ± 41.15	463.44 ± 39.63	522.42 ± 37.52
Mean 1st Third	470.95 ± 68.48	380.21 ± 33.01	426.46 ± 36.73	384.09 ± 50.95	504.57 ± 39.58	488.86 ± 45.63	515.91 ± 45.61
Mean 2nd Third	489.10 ± 76.81	372.51 ± 42.27	422.48 ± 35.99	422.86 ± 50.65	553.46 ± 47.41	490.66 ± 42.86	533.80 ± 42.17
Mean 3rd Third	462.42 ± 74.98	376.67 ± 33.83	403.40 ± 30.11	398.41 ± 60.67	525.03 ± 50.21	410.33 ± 44.48	517.21 ± 36.91
Median F0	479.84 ± 72.48	373.58 ± 34.70	418.80 ± 35.22	407.77 ± 52.70	537.13 ± 43.74	479.57 ± 43.94	525.61 ± 37.28
Frequency Range	105.36 ± 54.05	88.73 ± 44.02	69.52 ± 19.04	88.73 ± 38.16	137.91 ± 58.94	140.03 ± 53.94	93.69 ± 49.81
**Temporal parameters (s)**							
Duration	1.3849 ± 1.4625	3.4262 ± 2.3277	3.1106 ± 1.7148	0.5590 ± 0.2337	0.9723 ± 0.3626	0.3833 ± 0.1116	1.0090 ± 0.3865
Min F0 Loc	0.5588 ± 0.4703	0.6066 ± 0.3744	0.9213 ± 0.2165	0.2112 ± 0.3987	0.3308 ± 0.4653	0.9292 ± 0.2531	0.4979 ± 0.4829
Peak F0 Loc	0.4411 ± 0.2849	0.4184 ± 0.4107	0.1473 ± 0.2299	0.5319 ± 0.1471	0.5432 ± 0.2081	0.2938 ± 0.1045	0.5216 ± 0.2837
Time Min/Max	0.6291 ± 0.2290	0.5552 ± 0.2854	0.8370 ± 0.1923	0.5380 ± 0.1475	0.6169 ± 0.1680	0.6616 ± 0.1635	0.6090 ± 0.2547
**Shape and contour parameters**							
COFM	0.0196 ± 0.0099	0.0236 ± 0.0118	0.0143 ± 0.0050	0.0145 ± 0.0062	0.0243 ± 0.0102	0.0198 ± 0.0078	0.0185 ± 0.0102
Jitter Factor	2.1601 ± 1.1960	1.7501 ± 0.7013	1.1413 ± 0.5007	2.3389 ± 0.9497	2.4644 ± 1.0460	3.5508 ± 1.4778	1.8005 ± 0.8969
Frequency Variabilty Index	0.0527 ± 0.0551	0.0417 ± 0.0315	0.0170 ± 0.0083	0.0560 ± 0.0414	0.0624 ± 0.0405	0.1227 ± 0.0879	0.0309 ± 0.0302
Inflection Factor	0.2411 ± 0.1177	0.2712 ± 0.0951	0.2773 ± 0.1131	0.2105 ± 0.1250	0.2655 ± 0.1045	0.1188 ± 0.0690	0.2665 ± 0.1155
Start Slope	280.9714 ± 385.5385	28.9254 ± 129.8054	-1.0082 ± 65.0923	403.1685 ± 177.3596	382.0272 ± 288.4480	685.6907 ± 694.1020	192.2316 ± 197.8503
Middle Slope	-29.087 ± 148.0594	16.0784 ± 81.5588	-11.5420 ± 23.4030	39.5507 ± 112.1029	40.9863 ± 105.4892	-264.4057 ± 181.8868	-9.2500 ± 98.0554
Final Slope	-256.2098 ± 333.0340	-86.7713 ± 213.5505	-49.5427 ± 45.5595	-198.9493 ± 223.2615	-263.4847 ± 238.5296	-820.1854 ± 369.2074	-150.6676 ± 189.5794
