# Supplementary material for: Acoustic structure and information content of trumpets in female Asian elephants (Elephas maximus)
Source: PLoS One. 2021 Nov 23;16(11):e0260284. doi: 10.1371/journal.pone.0260284 (PMC8610244; doi:10.1371/journal.pone.0260284)
Supplement: S2 Table — (PDF) [file pone.0260284.s002.pdf]

S2 Table: Mean ± SD for all extracted acoustic parameters of the fundamental frequency of the higher frequency component of biphonic trumpets.

G0	all (117)	Chan Chun (20)	Dhibya (21)	Dipendra (11)	Saraswati (7)	Sona (11)	Sunder (47)
**Absolute frequency parameters (Hz)**							
Start F0	494.53 ± 71.47	418.99 ± 46.10	481.44 ± 37.03	461.51 ± 83.36	488.32 ± 56.58	486.82 ± 42.86	542.97 ± 61.98
Mid F0	515.06 ± 73.68	431.25 ± 74.80	455.32 ± 24.80	508.87 ± 61.44	561.37 ± 66.27	538.38 ± 34.03	566.50 ± 40.17
Finish F0	487.36 ± 82.07	413.90 ± 67.29	445.85 ± 38.27	484.18 ± 67.99	517.20 ± 78.14	427.40 ± 97.75	547.50 ± 52.68
Minimum F0	457.64 ± 72.66	379.71 ± 43.83	423.02 ± 24.35	452.06 ± 71.13	464.92 ± 39.99	415.89 ± 84.39	516.27 ± 47.67
Maximum F0	545.44 ± 70.21	478.65 ± 64.24	496.22 ± 35.52	520.78 ± 69.56	580.83 ± 69.64	549.09 ± 38.12	595.50 ± 47.25
Mean F0	508.09 ± 69.13	430.92 ± 58.97	455.11 ± 23.26	493.82 ± 63.70	548.41 ± 58.00	505.29 ± 42.56	562.58 ± 38.23
Mean 1st Third	508.18 ± 68.04	432.48 ± 51.97	467.92 ± 26.90	479.91 ± 74.33	534.57 ± 50.48	521.85 ± 39.50	557.86 ± 47.70
Mean 2nd Third	514.23 ± 73.01	430.83 ± 72.67	454.90 ± 23.57	503.70 ± 58.36	561.84 ± 68.35	532.57 ± 35.25	567.31 ± 38.89
Mean 3rd Third	501.48 ± 78.45	429.47 ± 67.22	442.31 ± 27.76	496.54 ± 67.84	550.24 ± 65.29	458.68 ± 75.71	562.47 ± 45.36
Median F0	510.96 ± 70.63	432.40 ± 66.19	454.08 ± 22.63	497.69 ± 62.87	556.93 ± 63.47	519.39 ± 40.61	564.08 ± 37.15
Frequency Range	87.80 ± 52.66	98.94 ± 45.30	73.20 ± 35.23	68.73 ± 37.35	115.91 ± 68.89	133.20 ± 86.06	79.23 ± 46.34
**Temporal parameters (s)**							
Duration	1.7191 ± 1.7078	3.7128 ± 2.1255	3.0618 ± 1.7518	0.5425 ± 0.2631	1.2573 ± 0.3928	0.3451 ± 0.0947	0.9364 ± 0.3542
Min F0 Loc	0.5770 ± 0.4476	0.5272 ± 0.4158	0.7993 ± 0.2847	0.3164 ± 0.4615	0.7143 ± 0.4880	0.7133 ± 0.4604	0.5074 ± 0.4722
Peak F0 Loc	0.3956 ± 0.3138	0.3263 ± 0.3326	0.2113 ± 0.3015	0.5356 ± 0.2577	0.4620 ± 0.2150	0.3900 ± 0.2239	0.4662 ± 0.3219
Time Min/Max	0.6363 ± 0.2422	0.5764 ± 0.2826	0.7649 ± 0.1695	0.5568 ± 0.1982	0.5282 ± 0.2168	0.6531 ± 0.1910	0.6350 ± 0.2595
**Shape and contour parameters**							
COFM	0.0173 ± 0.0093	0.0268 ± 0.0108	0.0152 ± 0.0066	0.0123 ± 0.0054	0.0193 ± 0.0098	0.0189 ± 0.0123	0.0147 ± 0.0066
Jitter Factor	1.6404 ± 0.9649	1.6098 ± 0.5615	1.1457 ± 0.5286	1.9326 ± 0.8052	1.4662 ± 0.5342	3.2698 ± 1.8558	1.4507 ± 0.6004
Frequency Variabilty Index	0.0380 ± 0.0579	0.0530 ± 0.0480	0.0215 ± 0.0225	0.0262 ± 0.0242	0.0343 ± 0.0309	0.1212 ± 0.1323	0.0229 ± 0.0329
Inflection Factor	0.2923 ± 0.1274	0.2568 ± 0.0838	0.3409 ± 0.1064	0.2642 ± 0.1553	0.2630 ± 0.1187	0.1784 ± 0.1052	0.3234 ± 0.1327
Start Slope	163.2510 ± 306.5249	57.8439 ± 140.9107	-29.8852 ± 80.9084	310.3661 ± 265.7199	248.5326 ± 278.7305	674.9262 ± 476.3816	127.5135 ± 242.9349
Middle Slope	-15.4589 ± 113.9717	-2.3359 ± 27.3121	-10.1828 ± 26.0363	26.4662 ± 141.0051	7.9228 ± 84.6256	-167.8330 ± 230.7547	-1.0333 ± 94.6870
Final Slope	-126.9515 ± 281.1756	-35.9360 ± 90.7351	-10.7473 ± 44.7755	-103.2118 ± 151.3554	-118.7230 ± 107.9545	-724.9463 ± 585.6152	-84.4279 ± 129.5025
